# Upregulated Circular RNA KIF4A Promotes Cell Migration and Invasion by Regulating MicroRNA-144-3p/EZH2 Axis in Gastric Cancer

**DOI:** 10.1155/2022/3985621

**Published:** 2022-04-15

**Authors:** Honglin Yan, Linyu Han, Na He, Rong Li, Shuixiang He

**Affiliations:** ^1^Department of Gastroenterology, The First Affiliated Hospital of Xi'an Jiaotong University, Xi'an, 710061 Shaanxi, China; ^2^Department of Gastroenterology, The First Affiliated Hospital of Xi'an Medical University, Xi'an, 710077 Shaanxi, China; ^3^Department of Pathology, The Affiliated Baoji Hospital of Xi'an Medical University, Baoji, 721006 Shaanxi, China; ^4^Department of Ophthalmology, The First Affiliated Hospital of Xi'an Medical University, Xi'an, 710077 Shaanxi, China

## Abstract

Accumulating evidence has shown that circular RNAs (circRNAs) serve a critical regulatory role in various human cancers, including gastric cancer (GC), and in this study, we aimed to explore the functions of circKIF4A in the progression of GC. Our findings demonstrated that circKIF4A was highly expressed in both GC tissues and cell lines, and high intratumoral circKIF4A expression predicted a poor prognosis in GC patients. In vitro gain- and loss-of-function assays indicated that circKIF4A knockdown suppressed the proliferation, migration, invasion, and EMT of GC cells, while these malignant behaviors were enhanced by circKIF4A overexpression. Mechanistically, we found that circKIF4A was mainly located in the cytoplasm, could directly interact with microRNA- (miR-) 144-3p, and functions as a miRNA sponge to regulate EZH2 expression in GC cells. miR-144-3p inhibition or EZH2 restoration largely blocked the effects of circKIF4A knockdown on the malignant behaviors of GC cells. This study indicated that circKIF4A can efficiently sponge miR-144-3p to promote the malignant behaviors of GC cells and may provide a potential biomarker and therapeutic target for GC management.

## 1. Introduction

Gastric cancer (GC) is a common malignancy of the digestive system, and it ranks as the fourth leading cause of cancer-related deaths around the world [1]. More than half of GC cases are diagnosed in East Asia [2]. Surgical resection and chemoradiotherapy are the main therapeutic methods for GC; however, the 5-year overall survival of GC patients with distant metastasis remains dismal [3]. Therefore, it is urgent to explore the mechanisms related to GC progression and metastasis.

Circular RNAs (circRNAs) are a very interesting class of endogenous noncoding RNAs featured by a covalently closed continuous loop without a 5′ cap and a 3′ poly A tail [4]. circRNAs often exhibit tissue-specific or developmental-stage-specific expression, and they can serve as crucial regulators of diverse physiological and pathological processes of organisms [5]. Recently, circRNAs are rapidly coming to the fore as diagnostic biomarkers and therapeutic targets in a wide variety of human cancers [6]. hsa_circ_0007255 (circKIF4A) is located at chrX: 69549254–69553539 and is upregulated in triple-negative breast cancer [7]. In the current study, we investigated the functions of circKIF4A in the progression of GC and identified a new regulatory mechanism.

## 2. Materials and Methods

### 2.1. Patients and Tissue Samples

In total, 107 pairs of GC tissues and adjacent noncancerous tissues (≥3 cm away from the tumor) were collected from patients who underwent surgical resection at hospital. The clinicopathological parameters of these patients are listed in Table 1. All patients did not receive any therapy before surgery. All tissue samples were immediately frozen in liquid nitrogen and stored at −80°C. The use of human tissues for this study has been approved by the Ethics Committee of the hospital. Written informed consents were obtained from all patients.

### 2.2. Cell Culture and Transfection

GC cell lines (SGC-7901, BGC-823, MKN-45, HGC-27) and a normal gastric epithelial cell line GES-1 were cultured in Dulbecco's modified Eagle's medium (DMEM; HyClone, Logan City, UT, USA) containing 10% fetal bovine serum (FBS; Sigma-Aldrich, St. Louis, MO, USA) and 100 mg/ml penicillin/streptomycin at 37°C in a humidified incubator with 5% CO_2_.

The small interfering RNA (siRNA) targeting circKIF4A (si-circKIF4A), the negative control siRNA (si-NC), miR-144-3p mimics, negative control (NC) mimics, miR-144-3p inhibitor, and negative control (NC) inhibitor were obtained from Guangzhou RiboBio Co., Ltd. (Guangzhou, China). circKIF4A or EZH2 cDNA was amplified and subcloned into the pcD-ciR vector (Geneseed Biotech Inc., Guangzhou, China) to construct the overexpression plasmid. The empty vector served as negative control. When the confluence reached 80%, cells were transiently transfected with the designated molecular products using Lipofectamine 2000 (Invitrogen, Carlsbad, CA, USA).

### 2.3. RT-qPCR Analysis

Total RNA was extracted using TRIzol reagent (Invitrogen). RNA was reverse transcribed to cDNA by using the PrimeScript™ RT Reagent Kit (TaKaRa). qPCR assay was then performed using a SYBR Green PCR Kit (TaKaRa) on an iCycler iQ™ Real-Time PCR Detection System (Bio-Rad Laboratories, Hercules, CA, USA). Relative gene expression was analyzed using the 2^−*ΔΔ*Ct^ method [8]. GAPDH or U6 was used as an internal control.

### 2.4. MTT Assay

Cells (2 × 10^3^ cells/well) were seeded into 96-well plates. At 0, 24, 48, and 72 h, 20 *μ*l of MTT dye (5 mg/ml; Sigma-Aldrich) was added to each well and the cells were incubated at 37°C for additional 4 h. After discarding the supernatant, the remaining formazan precipitates were dissolved in DMSO (Sigma-Aldrich). The absorbance of each well was measured at 570 nm on an ELx808 microplate reader (BioTek Instruments, Inc., Winooski, VT, USA).

### 2.5. Transwell Assay

Cells (5 × 10^4^ cells/well) suspended in 200 *μ*l of serum-free medium were added to the upper chambers of transwell plates (8 *μ*m pore size; BD Biosciences, Franklin Lakes, NJ, USA) with or without Matrigel coating. 500 *μ*l of medium containing 10% FBS was added to the lower chamber. After incubation for 48 h, the cells on the lower surface were fixed with 4% paraformaldehyde, stained with 0.1% crystal violet, and photographed under a microscope.

### 2.6. Western Blot Analysis

Total protein was extracted with RIPA lysis buffer (Beyotime, Shanghai, China). Protein extractions were separated by SDS-PAGE and transferred onto polyvinylidene fluoride (PVDF) membranes (Sigma-Aldrich). After blocking with 5% nonfat milk, the membranes were incubated with specific primary antibodies at 4°C overnight, followed by incubation with the HRP-conjugated secondary antibody for 1 h at room temperature. After washes, the protein bands were visualized using an electrogenerated chemiluminescence kit (Pierce Biotechnology, Rockford, IL, USA). GAPDH was used as an internal control.

### 2.7. Dual-Luciferase Reporter Assay

The circKIF4A or EZH2 mRNA sequence containing the predicted miR-144-3p-binding sites was inserted into the psiCHECK-2 vector (Promega, Madison, WI, USA). The binding sites were mutated by the GeneTailor™ Site-Directed Mutagenesis System (Invitrogen). Cells were seeded into 24-well plates and cotransfected with the reporter plasmids and miR-144-3p mimics or NC mimics using Lipofectamine 2000. After 48 h, the luciferase activity was detected using the Dual-Luciferase Reporter Assay System (Promega).

### 2.8. Statistical Analysis

All statistical analysis was performed by GraphPad Prism 6.0 software (GraphPad Software Inc., San Diego, CA, USA) and SPSS 18.0 software (SPSS Inc., Chicago, IL, USA). Data were expressed as mean ± standard deviation (SD) of three repeated experiments. Student's *t*-test, chi square test, or one-way ANOVA followed by Tukey's post hoc test was used for statistical comparisons as required. Survival curves were generated using the Kaplan–Meier method and assessed with the log-rank test. All *P* values were two sided, and *P* < 0.05 was statistically significant.

## 3. Results

### 3.1. circKIF4A Is Upregulated in GC

**Figure 1:**
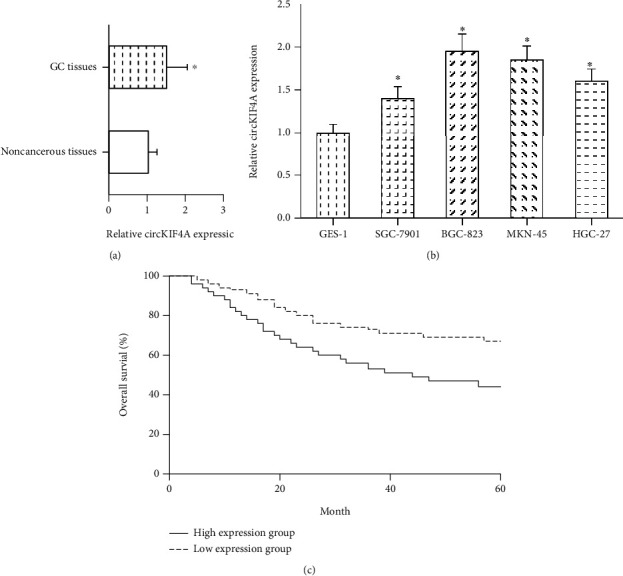
circKIF4A is upregulated in GC. (a) RT-qPCR analysis of circKIF4A expression in 107 pairs of GC tissues and adjacent noncancerous tissues. (b) RT-qPCR analysis of circKIF4A expression in GC cell lines and normal gastric epithelial cells. (c) The relationship between overall survival and circKIF4A expression in 107 GC patients. ^∗^*P* < 0.05 vs. noncancerous tissues or GES-1 cells.

We first observed that the expression of circKIF4A was significantly increased in human GC tissues, compared to that in adjacent noncancerous tissues (Figure 1(a)). In addition, compared with normal gastric epithelial cell line GES-1, circKIF4A was significantly upregulated in GC cell lines (SGC-7901, BGC-823, MKN-45, and HGC-27) (Figure 1(b)).

Based on the mean value of intratumoral circKIF4A expression, these 107 GC patients were allocated into a high expression group (*n* = 51) and low expression group (*n* = 56). We observed that high circKIF4A expression was significantly associated with larger tumor size (*P* = 0.018) and advanced TNM stage (*P* = 0.030) in GC patients (Table 1). Moreover, we noted that GC patients with high circKIF4A expression exhibited poorer overall survival compared with those with low circKIF4A expression (*P* = 0.016; Figure 1(c)).

### 3.2. circKIF4A Knockdown Inhibits GC Cell Proliferation, Migration, and Invasion

**Figure 2:**
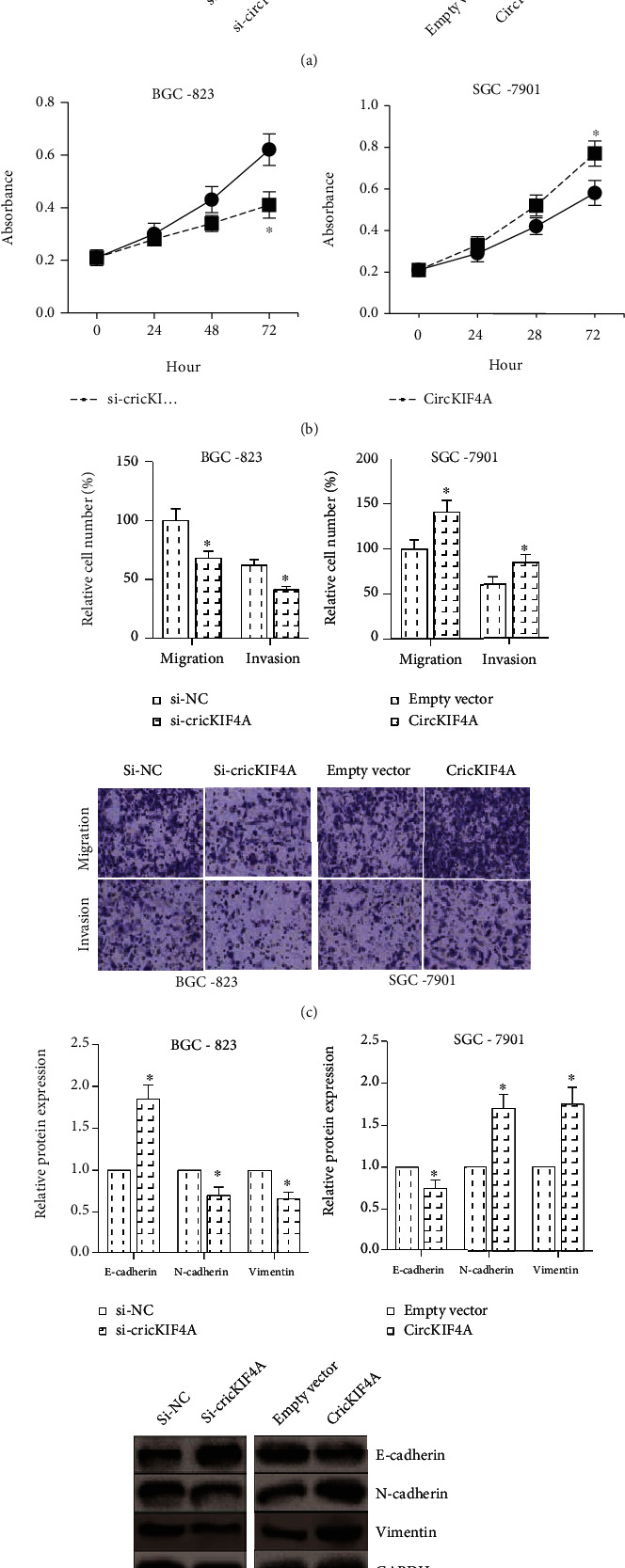
circKIF4A knockdown inhibits GC cell proliferation, migration, and invasion. (a) RT-qPCR analysis of circKIF4A expression in GC cells after transfection with si-circKIF4A/circKIF4A overexpression plasmid. (b) MTT assay for the proliferation of GC cells after circKIF4A knockdown/overexpression. (c) Transwell assay for the migration and invasion of GC cells after circKIF4A knockdown/overexpression. (d) Western blot analysis of E-cadherin, N-cadherin, and Vimentin protein expression in GC cells after circKIF4A knockdown/overexpression. ^∗^*P* < 0.05 vs. si-NC or empty vector-transfected cells.

Gain- and loss-of-function assays were further carried out to verify the functional role of circKIF4A in GC. As demonstrated in Figure 2(a), after transfection with si-circKIF4A, circKIF4A expression was significantly decreased in BGC-823 cells. We also overexpressed circKIF4A in SGC-7901 cells. MTT assay showed that circKIF4A knockdown notably decreased the proliferation rate of BGC-823 cells (Figure 2(b)), and transwell assay indicated that the migration and invasion abilities of BGC-823 cells were remarkably suppressed by circKIF4A knockdown (Figure 2(c)). circKIF4A overexpression obviously promoted the proliferation, migration, and invasion of SGC-7901 cells. EMT is a critical step of tumor metastasis [9]. circKIF4A knockdown led to the upregulation of E-cadherin and the loss of N-cadherin and Vimentin in BGC-823 cells, implying the inhibition of EMT, while the EMT of SGC-7901 cells was markedly enhanced by circKIF4A overexpression, as demonstrated by western blot analysis (Figure 2(d)).

### 3.3. circKIF4A Directly Binds to miR-144-3p in GC

**Figure 3:**
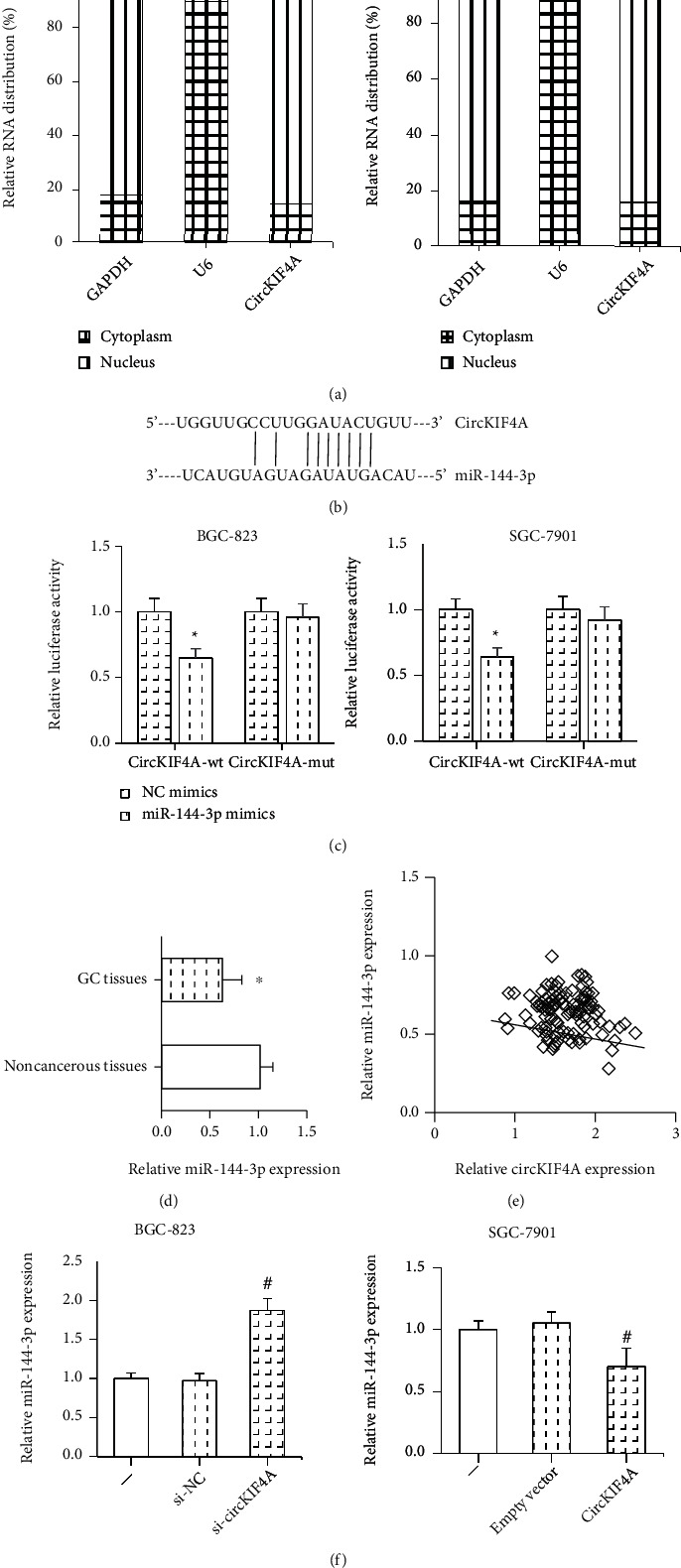
circKIF4A directly binds to miR-144-3p in GC. (a) The subcellular distribution of circKIF4A in GC cells. (b) The predicted binding sites between circKIF4A and miR-144-3p. (c) Dual-luciferase reporter assay for the binding relation between circKIF4A and miR-144-3p. (d) RT-qPCR analysis of miR-144-3p expression in 107 pairs of GC tissues and adjacent noncancerous tissues. (e) The correlation between circKIF4A and miR-144-3p expression in GC tissues. (f) RT-qPCR analysis of miR-144-3p expression in GC cells after circKIF4A knockdown/overexpression. ^∗^*P* < 0.05 vs. NC mimics-transfected cells or noncancerous tissues; ^#^*P* < 0.05 vs. si-NC or empty vector-transfected cells.

We further confirmed that circKIF4A was predominantly located in the cytoplasm of BGC-823 and SGC-7901 cells (Figure 3(a)), indicating that it may function as microRNA sponges in GC. By using the starBase online software (http://starbase.sysu.edu.cn/index.php), we noted that circKIF4A formed complementary base pairing with miR-144-3p (Figure 3(b)). Dual-luciferase reporter assay was then performed to confirm the binding relation, and we found that the luciferase activity of circKIF4A-wt but not circKIF4A-mut was overtly inhibited in BGC-823 and SGC-7901 cells after cotransfection with miR-144-3p mimics (Figure 3(c)). Furthermore, miR-144-3p expression was significantly lower in GC tissues than in adjacent noncancerous tissues (Figure 3(d)), and an inverse correlation was found between the expression of circKIF4A and miR-144-3p in GC tissues (Figure 3(e)). Also, miR-144-3p expression was distinctly inhibited by circKIF4A overexpression in SGC-7901 cells and was increased by circKIF4A knockdown in BGC-823 cells (Figure 3(f)).

### 3.4. miR-144-3p Inhibition Abates the Effects of circKIF4A Knockdown in GC Cells

**Figure 4:**
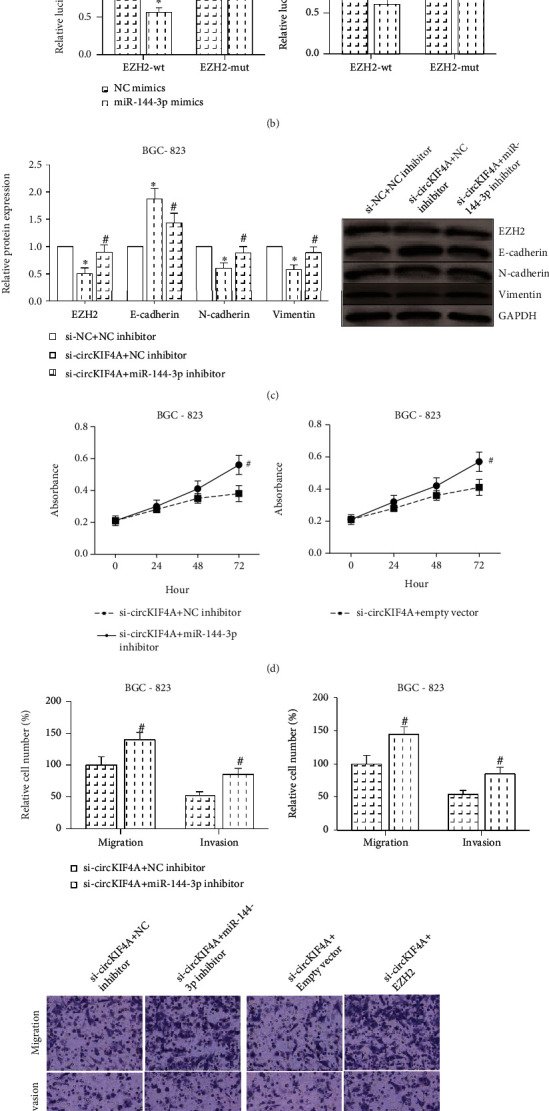
miR-144-3p inhibition abates the effects of circKIF4A knockdown in GC cells. (a) The predicted binding sites between EZH2 mRNA and miR-144-3p. (b) Dual-luciferase reporter assay for the binding relation between EZH2 mRNA and miR-144-3p. (c) Western blot analysis of EZH2, E-cadherin, N-cadherin, and Vimentin protein expression in GC cells with circKIF4A knockdown after miR-144-3p inhibition. (d) MTT assay for the proliferation of GC cells with circKIF4A knockdown after miR-144-3p inhibition/EZH2 restoration. (e) Transwell assay for the migration and invasion of GC cells with circKIF4A knockdown after miR-144-3p inhibition/EZH2 restoration. ^∗^*P* < 0.05 vs. NC mimics or si-NC-transfected cells; ^#^*P* < 0.05 vs. NC inhibitor or empty vector-transfected cells.

According to TargetScan software (http://www.targetscan.org), we noted that the 3′UTR of EZH2 mRNA possessed the potential binding sites for miR-144-3p (Figure 4(a)). The luciferase activity of EZH2-wt but not EZH2-mut was remarkably repressed in BGC-823 and SGC-7901 cells after cotransfection with miR-144-3p mimics (Figure 4(b)). In addition, as shown in Figure 4(c), the decreased EZH2 protein expression in BGC-823 cells with circKIF4A knockdown was notably rescued by inhibition of miR-144-3p, accompanied by the promotion of EMT. What is more, the suppression of malignant behaviors in BGC-823 cells caused by circKIF4A knockdown could be largely restored by ectopic expression of EZH2 or inhibition of miR-144-3p (Figures 4(d) and 4(e)).

## 4. Discussion

At present, GC remains a heavy burden on human society. Tumor progression is a multistage, multifactorial pathological process, and GC is also caused by a gradual accumulation of multiple genetic and epigenetic aberrations [10]. With the continuous improvement of molecular biology techniques, a large number of circRNAs have been identified to serve a regulatory role in GC. Due to their great abundance and high stability, circRNAs exhibit a great potential to be exploited as biomarkers for GC [11, 12].

In the study, we noted that circKIF4A was highly expressed in both GC tissues and cell lines, and high intratumoral circKIF4A expression predicted a poor prognosis in GC patients, implying its clinical value. We then performed a series of gain- and loss-of-function assays to verify the functional role of circKIF4A in GC, and the results showed that circKIF4A knockdown inhibited, while circKIF4A overexpression promoted the malignant behaviors, such as proliferation, migration, and invasion, of GC cells. Metastasis is a leading cause of GC-related deaths, and EMT confers metastatic properties upon GC cells [13]. Inhibition or reversal of EMT seems to be an effective strategy to treat GC patients [14]. Our study confirmed that circKIF4A overexpression enhanced EMT in GC cells.

In recent years, more and more articles demonstrated that circRNAs could competitively suppress the functions of miRNAs through serving as molecular sponges [15]. The circRNA-miRNA-mRNA axes are frequently involved in human diseases [16]. miR-144-3p is mainly considered as a tumor suppressor in many cancers, such as cervical cancer and colorectal cancer [17, 18]. Also, miR-144-3p is downregulated in the plasma of GC patients and suppresses GC progression by inhibiting EMT [19, 20]. This study showed that circKIF4A was mainly located in the cytoplasm and could directly interact with miR-144-3p and negatively regulate its expression in GC cells. EZH2 is a well-known oncogene in many cancers, and its overexpression is correlated with poor prognosis in GC patients [21]. EZH2 also led to the acquisition of EMT phenotype of GC cells [22]. This study confirmed that EZH2 is a direct target of miR-144-3p in GC. Rescue analysis further demonstrated that miR-144-3p inhibition or EZH2 restoration could largely block the effects of circKIF4A knockdown on the malignant behaviors of GC cells.

In conclusion, the results of our study indicated that circKIF4A is overexpressed in human GC samples, and it can efficiently sponge miR-144-3p to promote the malignant behaviors of GC cells. Our study may provide a potential biomarker and therapeutic target for GC management.

## Figures and Tables

**Table 1:** The relationship between clinicopathological characteristics and circKIF4A expression in 107 GC patients.

Characteristics	Total number (*n* = 107)	circKIF4A expression		*P* value
		Low (*n* = 56)	High (*n* = 51)	
Age (years)				0.360
≤55	49	28	21	
>55	58	28	30	
Gender				0.674
Male	61	33	28	
Female	46	23	23	
Tumor size (cm)				0.018
<5	65	40	25	
≥5	42	16	26	
Differentiation				0.240
Well+moderate	67	38	29	
Poor	40	18	22	
TNM stage				0.030
I-II	66	40	26	
III-IV	41	16	25	
Lymph node metastasis				0.118
Yes	63	29	34	
No	44	27	17	

## Data Availability

The data used to support the findings of this study are available from the corresponding author upon request.
